# Research on Multiphysics-Driven MEMS Safety and Arming Devices

**DOI:** 10.3390/mi15101194

**Published:** 2024-09-26

**Authors:** Xinyu Fan, Tengjiang Hu, Yifei Wang, Yulong Zhao, Zhongwang Tian, Wei Xue

**Affiliations:** 1 State Key Laboratory for Manufacturing Systems Engineering, Xi’an Jiaotong University, Xi’an 710049, China; fanxy@stu.xjtu.edu.cn (X.F.); zhaoyulong@xjtu.edu.cn (Y.Z.); 2 AVIC Xi’an Aeronautics Computing Technique Research Institute, Xi’an 710065, China; wangyifei990907@163.com; 3 Science and Technology on Electromechanical Dynamic Control Laboratory, Xi’an Institute of Electromechanical Information Technology, Xi’an 710065, China; tianzw129@163.com (Z.T.); nuc_xuewei@163.com (W.X.)

**Keywords:** MEMS, safety and arming (S&A) devices, multiphysics driven

## Abstract

As the core component of energy transfer in weapon system, safety and arming (S&A) devices affect the safety, reliability, and damage ability of the weapon. Micro-electromechanical systems (MEMS) S&A devices have been widely investigated for their smaller structure size, higher functional integration, and better smart functionality. This paper proposes the design of a multi-physics field-driven MEMS S&A device. The S&A mechanism is composed of a setback mechanism, a spin mechanism, and an electrothermal mechanism, achieving multiphysics-arming. With the coordination of the three mechanisms, the S&A device can produce a 1 mm displacement. The displacement generated allows the S&A device to switch between safety status and arming status. The unlock conditions and overload resistance of each mechanism are obtained by finite element simulation. Based on SOI wafers and silicon oxide wafers, the chips were fabricated and packaged. Several tests were carried out to verify the working condition and overload resistance of the S&A device. The result shows that under a voltage of 11 V and a rotation speed of 8000 r/min, with a size no more than 10 mm × 10 mm × 1.5 mm, the device works smoothly and can withstand an overload of 25,000 g.

## 1. Introduction

The safety-and-arming (S&A) device, as one of the core components of the weapon system, is applied to regulate the energy transfer process of the energy material. Specifically, it is used to detonate controlled explosions and prevent accidental explosions. The function of the S&A device significantly concerns the safety, reliability, and lethality of the weapon [1]. Thus, the development of high-performance S&A devices is of great importance in modern warfare and aligns with national strategic priorities.

A traditional mechanical S&A device consists of a spring-mass system, driven by a specific environmental inertia force. Due to its driven method and structure size, it is hard for the traditional S&A device to integrate into small-caliber ammunition (less than 35 mm in diameter). With the rapid development of micro-electromechanical systems (MEMS) technology, the MEMS S&A device has been widely investigated due to its advantage in smaller structure size, higher functional integration, and better smart functionality [2,3,4]. The features of MEMS S&A devices can be summarized as follows: (**a**). **Structure miniaturization**. For the MEMS S&A device, the size of the overall system is in the order of millimeters, while the size of the structure is in the order of micrometers. (**b**). **Smart control**. Based on driving principles, MEMS S&A devices mainly included inertial force driving [5,6,7,8], electro-thermal driving [9,10], pyrotechnic driving [11,12,13], and electromagnetic driving [14,15]. To be specific, in the case of the inertial force driving method, Jeong et al. [6] and Seok et al. [7] presented two miniature S&A devices driven by setback and centrifugal loads. For the electro-thermal driving method, Wang et al. [10] proposed an MEMS S&A device driven by an electro-thermal actuator, generating a displacement of 117 μm under a voltage of 11 V. As for the pyrotechnics driving method, Zhu et al. [12] designed a pyrotechnical micro-electromechanical system (PyroMEMS) S&A device, which uses C_6_H_2_(NO_2_)_3_OK/KClO_4_, the gas charge, to drive the moveable structure in the device. In terms of the electromagnetic driving method, Lv et al. [14,15] developed an electromagnetic-driven Sibased MEMS S&A device, which can produce a driving force of 270 mN under the condition of a distance of 0.1 mm and 8 V. By applying multiphysics driven method, smart control of the S&A device can also be achieved; (**c**). **Sequence integration**. In an MEMS S&A device, the micro energy material, micro actuator, and micro explosive train are integrated in one system, realizing functions such as information exchange, S&A control, and output selection [16].

This paper introduces a design of multiphysics-driven MEMS S&A devices, enabling multiphysics-arming, smart control, and high resistance to overload. This MEMS S&A device has dimensions of no more than 10 mm × 10 mm × 1.5 mm and operates at a drive voltage of 11 V. With the help of a setback mechanism and an electrothermal mechanism [17], the S&A devices achieve multiphysics-arming (inertial field and electro-thermal field) by inertia force and thermoelectric force. Under the effect of centrifugal force, a spin barrier (in spin mechanism) will produce a 1 mm displacement to turn to the arming position. Simulation and several tests are introduced to validate its functionality.

## 2. Modeling

### 2.1. Structure of the S&A Device

**Figure 1:**
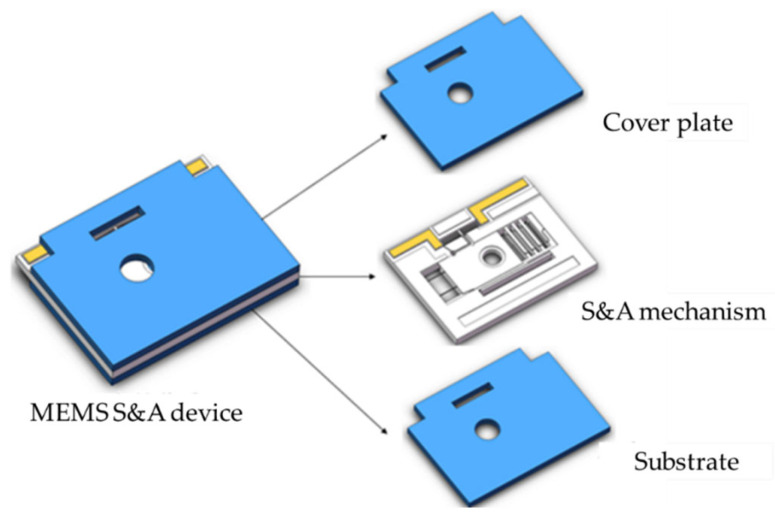
The structure of MEMS S&A device.

The device measures 6.0 × 8.0 × 1.3 mm and mainly consists of an S&A structure of SOI wafers (50 μm device layer, 3 μm buried layer and 400 μm handle layer, Okmetic, Vantaa, Finland), cover plate, and substrate of thick oxide layer silicon wafer (Okmetic, Vantaa, Finland), shown in Figure 1. The three components are bonded together with high-strength epoxy resin adhesive.

**Figure 2:**
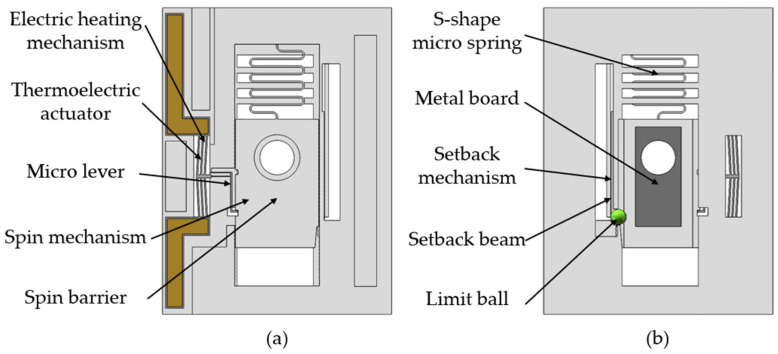
The structure of MEMS S&A mechanism: (**a**) the front of the S&A mechanism; (**b**) the back of the S&A mechanism.

The structure of S&A mechanism is shown in Figure 2, comprising a setback mechanism, a spin mechanism, and an electrothermal mechanism. These mechanisms are able to switch their status between safety and arming. When the three mechanisms are in the arming status simultaneously, the S&A device turns to arming status.

**Figure 3:**
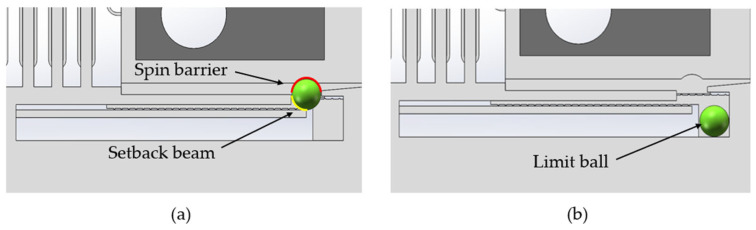
The structure of setback mechanism: (**a**) setback mechanism in safety status; (**b**) setback mechanism in arming status.

The setback mechanism comprises a setback beam and a limit ball (steel in material). As shown in Figure 3, in safe status, the ball is constrained between the spin barrier (in spin mechanism) and the setback beam. The spin barrier and the ball form a spring–mass system with the setback beam. Under the impact of the inertia force, the setback beam bends and the ball falls once the bending deflection reaches a certain threshold. The fall of the ball releases the lower part of the spin barrier, indicating that the setback mechanism is in the arming status.

**Figure 4:**
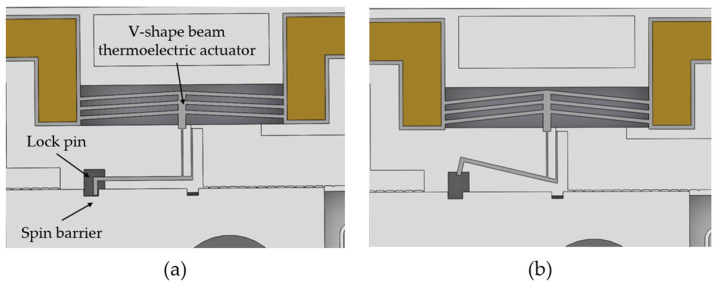
The structure of electrothermal mechanism: (**a**) electrothermal mechanism in safety status; (**b**) electrothermal mechanism in arming status.

The electrothermal mechanism comprises electrodes, a V-shape beam thermoelectric actuator, and a micro lever. As shown in Figure 4, in safe status, the end of the micro lever serves as a lock pin to constrain the spin barrier. When the current flow through the V-shape beam, a large amount of heat is produced. Due to thermal expansion, deformation is produced in the middle of the V-shape beam. The micro lever amplifies the deformation, thus, the lock pin releases the spin barrier, indicating the electrothermal mechanism is in the arming status.

**Figure 5:**
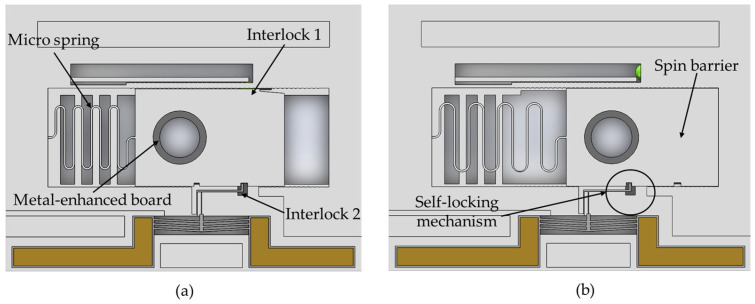
The structure of spin mechanism: (**a**) spin mechanism in safety status; (**b**) spin mechanism in arming status.

The spin mechanism comprises a micro-spring, spin barrier, and metal-enhanced board, as shown in Figure 5. The metal-enhanced board is installed within the spin barrier, featuring a hole that acts as an extra acceleration barrel. Under the impact of the centrifugal force, the barrier will move to the arming position, indicating the spin mechanism switches to arming status.

It is important to note that the spin mechanism is interlocked with the setback mechanism (interlock 1) and the electrothermal mechanism (interlock 2). Specifically, the setback mechanism interlocks with the spin mechanism by the restraint between the limit ball and the spin barrier; the electrothermal mechanism interlocks with the spin mechanism by the restraint between the lever’s ending and the spin barrier. Only if the two related mechanisms are in the arming status can the spin barrier move, indicating the spin mechanism turns to arming status.

The self-locking mechanism comprises the lock pin in the electrothermal mechanism and the spin barrier in the spin mechanism. After the spin barrier reaches the arming position, the electrothermal mechanism switches back to safe status (by turning off the voltage supply) to restrain the spin barrier again, achieving self-locking.

To sum up, in this design, the setback mechanism is supposed to turn to the arming status first. Subsequently, under the control of the electrothermal mechanism, the spin mechanism reaches the arming position, representing the device’s conversion from safe status to arming status. Additionally, with assistance from a self-locking mechanism, the device can maintain arming status without energy input.

### 2.2. Structure of the Detonation Device

**Figure 6:**
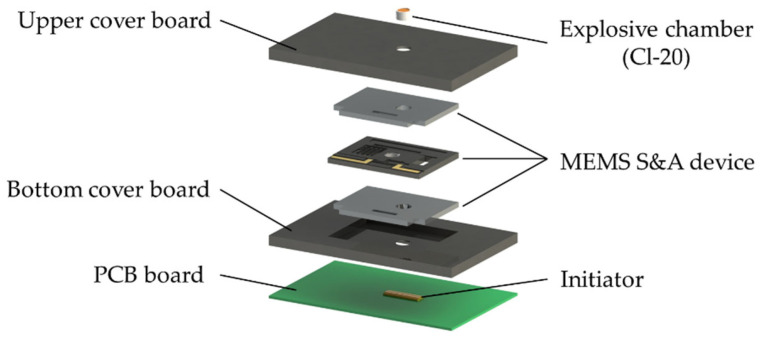
The structure of the detonating device.

The detonation device designed for the S&A device is shown in Figure 6. The detonation device comprises an initiator (Xi’an Institute of Electromechanical Information Technology, Xi’an, China), a pair of cover boards, an MEMS S&A device, and an explosive chamber with Hexanitrohexaazaisowurtzitane (CL-20) inside (Xi’an Institute of Electromechanical Information Technology, Xi’an, China). Under a surge voltage, the initiator creates a flyer that will detonate the energy material (CL-20) in the explosive chamber through the accelerating barrel [18,19].

**Figure 7:**
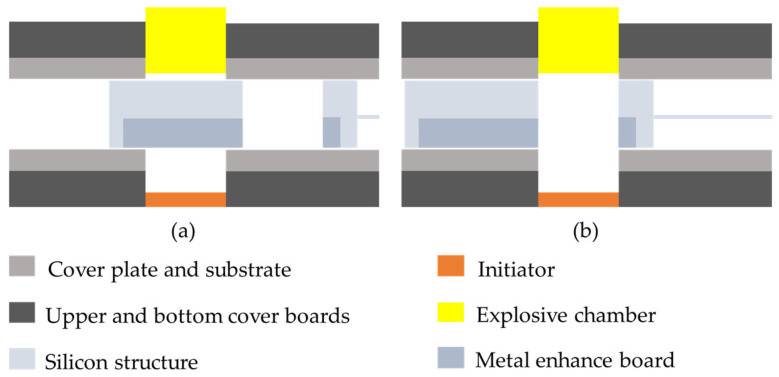
The working principle of the MEMS S&A device: (**a**) safety status, the acceleration barrel is blocked by the spin barrier; (**b**) arming status, the acceleration barrels are in alignment.

The working principle of the device is shown in Figure 7. In safe status, the acceleration barrel is obstructed by the spin barrier in the S&A device. In arming status, the acceleration barrel in the spin barrier and the one in the cover board are in alignment, enabling explosion.

## 3. Simulation

### 3.1. Simulation of the Setback Mechanism

The setback mechanism is a spring–mass system consisting of the setback beam and the limit ball. The setback beam can be simplified as a cantilever beam. According to the end deflection of cantilever beam based on material mechanics, the designed size of the setback mechanism is shown in Table 1.

**Figure 8:**
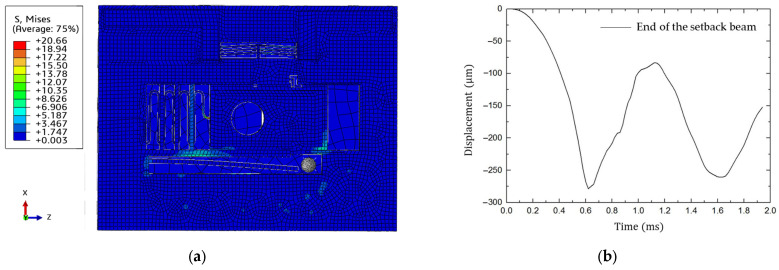
Dynamic simulation of setback mechanism (2 ms and 15,000 g): (**a**) stress nephogram of S&A mechanism; (**b**) deformation of the end of the setback beam.

A 2 ms and 15,000 g launching overload simulation is set up in Abaqus, presented in Figure 8.

**Figure 9:**
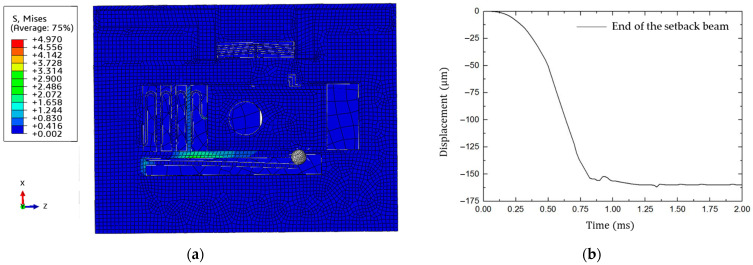
Dynamic simulation of setback mechanism (2 ms and 4000 g): (**a**) Stress nephogram of S&A mechanism; (**b**) Deformation of the end of the setback beam.

It demonstrates that the maximum stress of 20.66 MPa in the setback beam occurs at the fixed end, which is less than the fracture stress of silicon material. The maximum displacement, occurring at the fixed end of the setback beam as well, reaches 278.68 μm. The maximum displacement exceeds the minimum unlock displacement. To determine the minimum unlock load, a series of half-sine wave loads are applied. The applied loads last for 2 ms, with values set as 12,000 g, 10,000 g, 8000 g, 6000 g, and 4000 g, successively. The setback mechanism unlocks successfully for the first four groups but failed when the load decreases to 4000 g, as shown in Figure 9. According to Figure 9b, the limit ball reaches the maximum displacement 160 μm at 0.75 ms and remains in this state, indicating the minimum unlock load of the setback mechanism is determined to be at least 4000 g.

**Figure 10:**
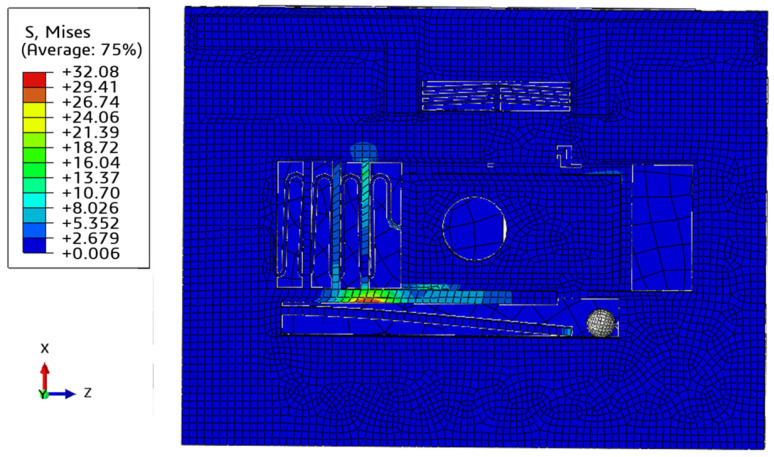
Dynamic simulation of setback mechanism (2 ms and 25,000 g).

To verify the axial anti-overload capability of the designed S&A device, a 2 ms and 25,000 g half-sine wave load is applied continuously. The results are shown in Figure 10. Since the axial overload is a downward force (opposite to X direction), the spin barrier will move downward and impact the setback beam, resulting in a severe stress concentration. However, the maximum stress observed is 32.08 MPa, which is much less than the yield limit of silicon material 170 MPa.

### 3.2. Simulation of the Spin Mechanism

The spin mechanism is a spring–mass system comprising a micro-S-shape spring, a spin barrier, and a metal-enhanced board. It is designed to reach the unlock position under a driving centrifugal force at a speed of 8000 r/min and with an eccentricity of 3 mm.

The micro-S-shape spring plays an important role in the spin mechanism. The mass of the metal-enhanced board impacts the total mass, which must be fully considered in the spring design. Besides, due to the influence of the frictional force and other resistance, the theoretical stiffness is supposed to be slightly smaller than its design stiffness. The theoretical stiffness (*K*_1_) and the design stiffness (*K*_2_) of the micro-S-shape spring may be calculated using Equation (1):

(1) $$\left\{\begin{array}{c} K_{1}=\frac{F}{x}=\frac{mw^{2}r}{x}\\ K_{2}=\frac{b^{3}hE}{n\left[16l^{3}+12R\left(2l^{2}\pi +8lR+R^{2}\pi \right)\right]} \end{array}\right.$$

where *K*_1_ is the design stiffness, *w* is the angular velocity, *r* is the eccentricity, *K*_2_ is the theoretical stiffness of the micro-spring, *b* is the width of the micro-spring, *h* is the thickness of the micro-spring, *E* is the Young’s modulus of silicon, *n* is the number of turns of the micro-spring, and *R* is the bending radius of the micro-spring. According to Equation (1), the basic parameters of the designed micro-S-shape are given in Table 2.

**Figure 11:**
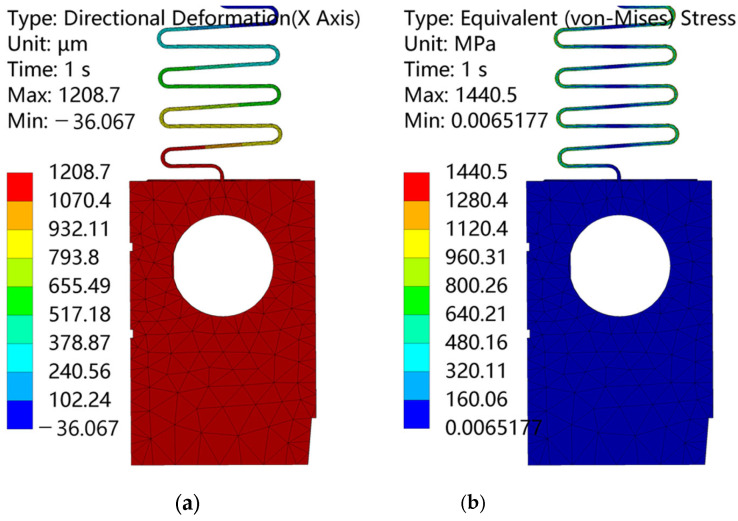
Simulation results of the spin mechanism (3 mm and 8000 r/min): (**a**) deformation of the spin mechanism; (**b**) stress of the spin mechanism.

Under an acceleration of 215 g produced by a centrifugal force (speed 8000 r/min, eccentricity 3 mm), a simulation of the spin system is conducted using ANSYS 2022 R1, which the result shows in Figure 11. It shows that the barrier reaches a maximum displacement of 1.21 mm, exceeding the design displacement of 1 mm. Also, the maximum stress observed is 1.44 GPa, which is less than the breaking strength. The 215 g acceleration simulated equals a 21,700 μN centrifugal force numerically. Combined with the displacement result, the simulation stiffness is found to be 21.48 N/m.

### 3.3. Simulation of the Electrothermal Mechanism

The electrothermal mechanism consists of a thermoelectric actuator, micro lever, and electrodes.

The basic component of the thermoelectric actuator is the V-shape silicon beam. Considering heat conduction, heat convection, and heat radiation, the temperature distribution of the V-shape beam can be determined based on a one-dimensional heat diffusion model as Equation (2) [19]:

(2) $$k_{s}\frac{\partial^{2}T(x,t)}{\partial x^{2}}+J^{2}\rho -Sk_{a}\frac{T(x,t)-T_{\infty }}{gh}=cD\frac{\partial T(x,t)}{\partial t}$$

where *k_s_* is the thermal conductivity of single-crystal silicon, *k_a_* is the thermal conductivity of air, *T_∞_* is the initial temperature, *J* is the current density, *ρ* is the resistivity of silicon, *c* is the specific heat capacity of silicon, *D* is the density of silicon, and *S* is the shape factor.

Based on the thermodynamic model, the steady-state temperature distribution of the beam is obtained. Using structural mechanics’ mechanical analysis method, the maximum thermal expansion deformation of the V-shape beam is found as Equation (3):

(3) $${\Delta y}_{max}=\frac{\alpha l\Delta T\sin\theta }{{\left(\frac{w}{l}\right)}^{2}+\sin^{2}\theta }$$

where *α* is the thermal expansion coefficient of silicon, ∆*T* is the average temperature of the beam, *θ* is the angle of the V-shaped beam, *w* is the width of the V-shape beam, $l=\frac{L}{2\cos\theta }$, and *L* is the length of the beam.

**Figure 12:**
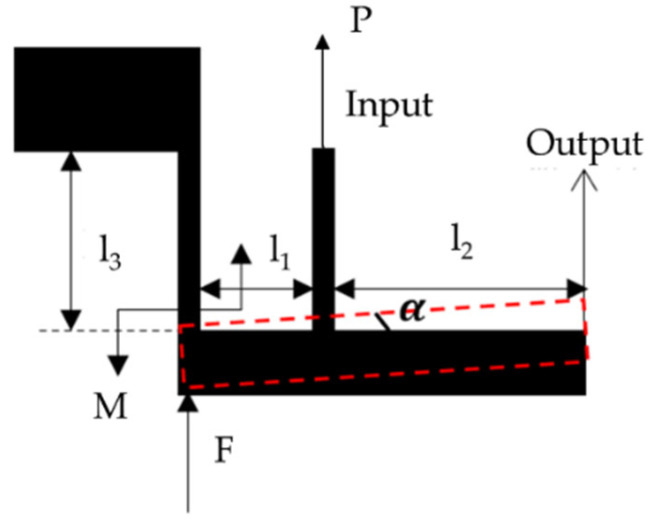
Micro lever structure diagram.

Furthermore, the thermal expansion coefficient of silicon is small, hence a displacement amplification mechanism is implemented to enhance the displacement produced by the actuator. A micro lever is adopted in this design, as shown in Figure 12. The micro-lever mechanism consists of two beams and a lever. One beam is for securing, and the other, connecting to the thermoelectric actuator, works as the input. The displacement amplification mechanism increases the input displacement with the amplification factor *A*, which is determined by the length of the lever arm’s ratio. The expression is: $A=\frac{l_{1}+l_{2}}{l_{1}}$.

The parameters of the electrothermal mechanism are shown in Table 3. It can be calculated that when the driving voltage is 11 V, the thermoelectric actuator achieves a maximum displacement of 8.46 μm and a maximum temperature of 644.63 K. The application of the displacement amplification mechanism amplifies the displacement by 10 times, reaching 84.6 μm.

**Figure 13:**
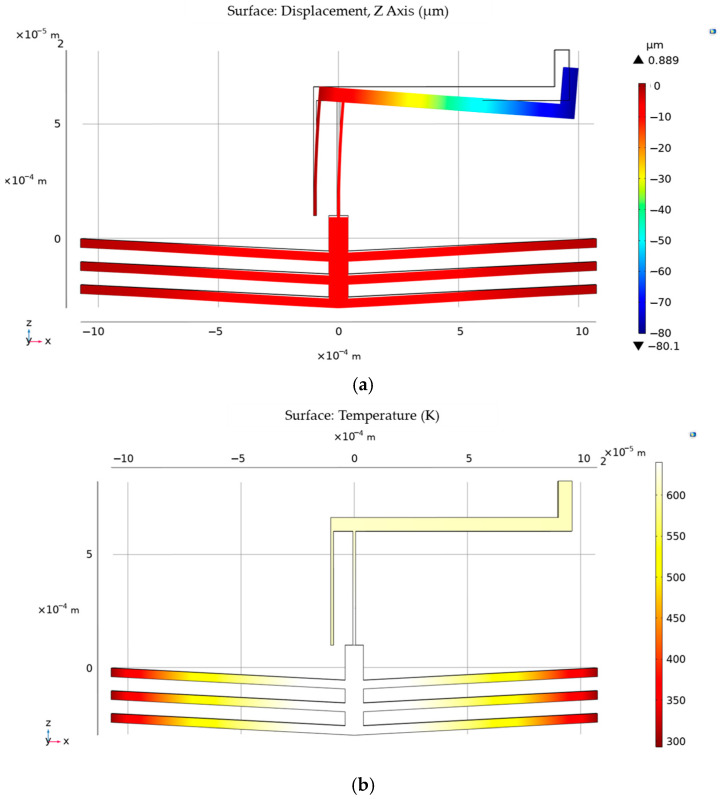
Simulation results of time-delay mechanism: (**a**) displacement of the thermoelectric actuator with micro lever; (**b**) temperature of the thermoelectric actuator with micro lever.

Simulations of the thermoelectric actuator with a micro lever are conducted separately using COMSOL 6.2, as shown in Figure 13. The maximum temperature reaches 644.17 K under the input voltage of 11 V, resulting in a variance of 0.07% from the theoretical value. In terms of displacement, the maximum displacement is 80.1 μm, exceeding the lock distance (62 μm). The discrepancy in displacement between the simulated result and the theoretical value (84.6 μm) can be attributed to additional heat loss through the amplification mechanism.

## 4. Fabrication

**Figure 14:**
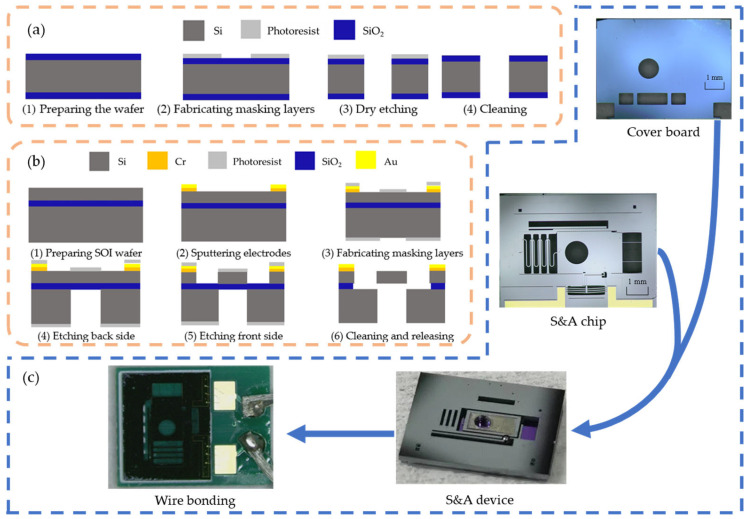
Fabrication process: (**a**) S&A chip processing process; (**b**) cover boards processing process; and (**c**) prototype of S&A device assembling process.

Four inch, (100) type SOI wafers are used for the fabrication of the S&A device. The wafer consists of a 50 μm device layer, a 3 μm buried layer, and a 400 μm handle layer. The fabrication process is shown in Figure 14a.

Four inch (N100) double-side polished silicon oxide wafers are used for the fabrication of the top-layer cover plate and the substrate. The thickness of the wafer is 400 μm, and the thickness of the oxide layer is 100 nm. The fabrication process is shown in Figure 14b.

The actual size of the S&A device chip is 6.0 mm × 8.0 mm, which is the same for the cover plate and substrate. Micro-assembling is completed by installing the limit ball and the metal-enhanced board into the device. The package process is completed through the subsequent wire bonding process. The process is shown in Figure 14c.

## 5. Testing

To determine whether the designed device can achieve the research objectives, several tests are required. Conducted at an ambient temperature, the unlock tests are carried out, including an unlock test of the setback mechanism, as well as an unlock test of the electrothermal mechanism and the spin mechanism. The function test is conducted subsequently to validate the S&A functionality of the device. Additionally, an overload test is performed to verify its reliability.

### 5.1. Unlock Test of Setback Mechanism

**Figure 15:**
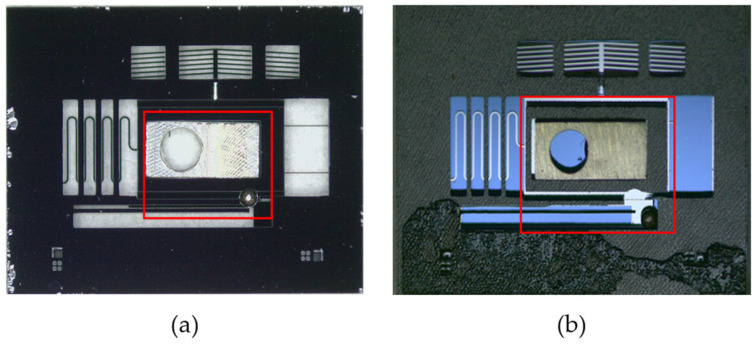
Setback mechanism test result (6 teeth): (**a**) S&A chip before test; (**b**) S&A chip after test.

The Machete Hammer experiment was introduced to validate whether the unlock performance of the setback mechanism satisfies the requirements. A calibration test was conducted to establish the correlation between the number of teeth and the overload, as shown in Table 4. According to Table 4 and the actual launch environment, the unlock test was carried out under the overload of six teeth (around 10,000 g) and seven teeth (around 15,000 g). It shows that the setback mechanism unlocks successfully when the overload is six teeth and seven teeth, as shown in Figure 15. The result confirms that the setback mechanism meets the requirements in the launch environment.

### 5.2. Unlock Test of Electrothermal Mechanism and Spin Mechanism

In the designed arming process, the spin mechanism is intended to work in coordination with the electrothermal mechanism. Thus, the electrothermal mechanism is supposed to be tested separately before the unlock test of both mechanisms.

**Figure 16:**
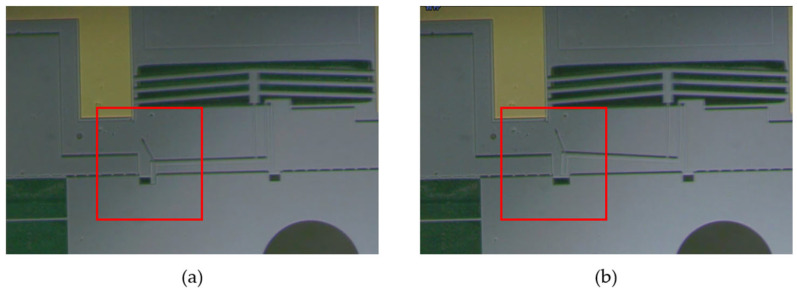
Electrothermal actuator test result: (**a**) electrothermal mechanism without the input voltage; (**b**) electrothermal mechanism under the input voltage.

The designed electrothermal mechanism controls the output displacement of the thermoelectric actuator by modulating the voltage. To test the output displacement of the electrothermal mechanism, a test platform is set up, comprising a DC voltage source, microscope, test sample, and display screen. The test results are shown in Table 5 and Figure 16. It shows that the electrothermal mechanism unlocks successfully under an 11 V voltage input, generating an output displacement of 71.25 μm (exceeding the length of the lock pin) and consuming 0.77 W power.

**Figure 17:**
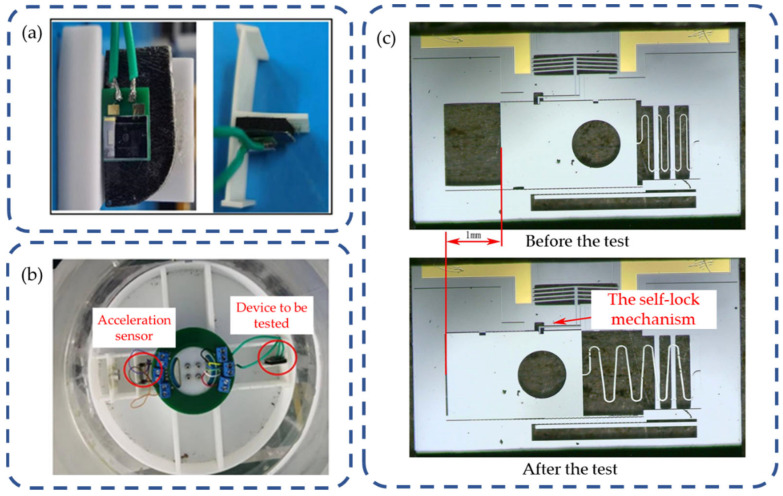
Unlock test of electrothermal mechanism and spin mechanism: (**a**) device to be tested; (**b**) principles of centrifuge testing; (**c**) test result.

For the unlock test of electrothermal mechanism and spin mechanism, a test platform consisting of a centrifuge and a laptop are set up to verify the reliability of the coordination. The test principle is shown in Figure 17b. In the actual launch situation, with an eccentricity of 3 mm, the MEMS S&A device experiences a 215 g acceleration at a rotation speed of 8000 r/min. To simulate the real load condition, the real-time data of the sensor is supposed to reach 103 g in the test.

The test result is shown in Figure 17c. When the real-time data of acceleration is low, the S-type micro-spring deforms under the effect of centrifugal force. This deformation causes a displacement in the spin barrier, yet it does not reach the designated position. When the real-time data of acceleration reaches 100 g, the barrier meets the designated position. Upon deactivating the DC power, the barrier is locked by the lock pin on the end of the electrothermal mechanism. The test result verifies that the spin mechanism and the electrothermal mechanism are able to coordinate the arming action under a rotation speed of 8000 r/min with an output arming displacement of 1 mm, realizing their function successfully.

### 5.3. Test of the Safe and Arming Functions

**Figure 18:**
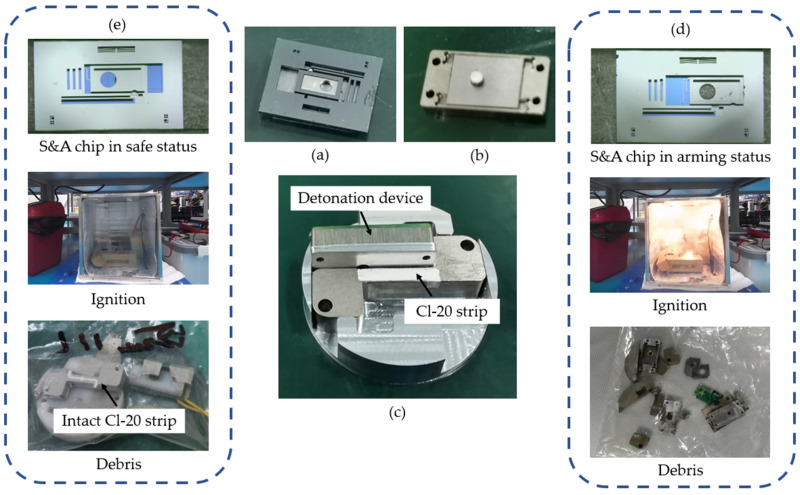
The explosion test: (**a**) S&A chips; (**b**) the detonation device, refers to Figure 6; (**c**) the explosion equipment; (**d**) the arming group; and (**e**) the safe group.

In a function test, the safety and arming function of the S&A device are tested separately. The detonation device, as shown in Figure 18b, is installed in the explosion equipment and connected to a CL-20 strip, as shown in Figure 18c. A 47 μF capacitance charged by a 25 V DC source is provided to drive the initiator. The result is shown in Figure 18d,e. In the arming group, the CL-20 strip is detonated successfully, with a loud sound and a clear flash. In contrast, for the safe group, a very slight flash and a much lower sound are produced, indicating the initiator is detonated successfully. Meanwhile, the CL-20 strip remains intact, which means the S&A device blocks the explosion successfully.

### 5.4. Overload Resistance Test

To verify whether the S&A device can stand a 25,000 g overload, the Machete Hammer experiment is introduced to test its overload resistance capability. The packaged S&A device is placed in the safety system, which is set in the test tool subsequently. Polyurethane cushion gaskets are added above and below the safety system to reduce the impact of the external load.

**Figure 19:**
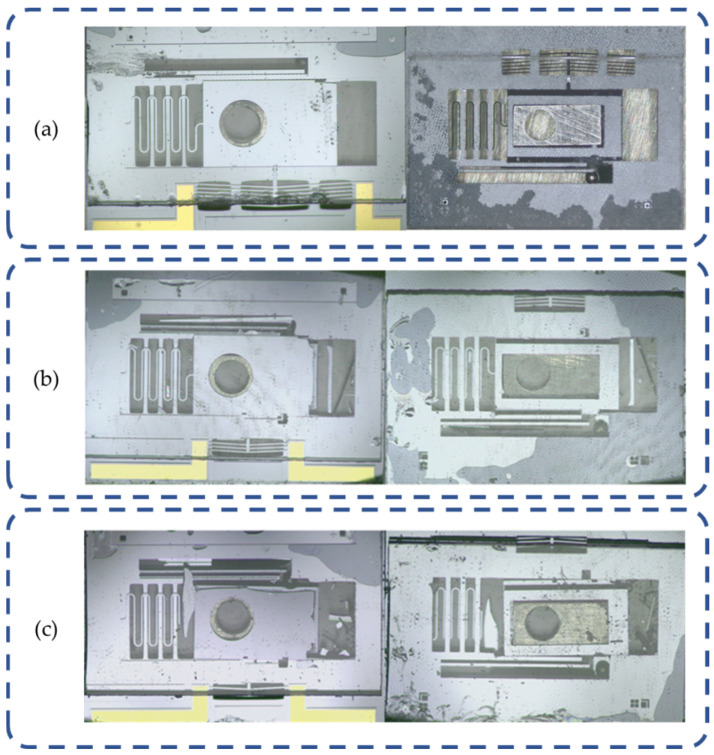
Overload resistance test result: (**a**) 9 teeth result; (**b**) 10 teeth result; and (**c**) 11 teeth result.

The hammering test was initiated with the ratchet turning to nine teeth, and the corresponding result is shown in Figure 19a. It demonstrates that the interior structures maintain intact, indicating the nine teeth load (approximately 26,000 g, according to Table 4) is successfully withstood. Based on the result, further tests are carried with loads of 10 teeth (approximately 34,000 g) and 11 teeth (approximately 42,000 g), and the result is shown in Figure 19b,c. The result indicates that under a load of 10 teeth, the setback mechanism unlocks and the micro lever as well as the electric heating mechanism stay intact. However, under a load of 11 teeth, the interior structure is damaged severely, leading to a failure of the S&A device. In summary, the designed S&A device has an overload resistance capability of 25,000 g, and the critical load should be slightly less than 33,000 g, according to Table 4.

## 6. Conclusions

In this paper, a multiphysics-driven MEMS S&A device with a controllable time-delay is designed, consisting of an S&A mechanism, cover plate, and substrate. The S&A mechanism comprising a setback mechanism, spin mechanism, and electrothermal mechanism, achieving multiphysics-arming (inertial field and electro-thermal field) by inertia force and thermoelectric force. The overall size of the security device is no more than 10 mm × 10 mm × 1.5 mm. Finite element simulation suggests that the minimum unlock load for the setback mechanism is 4000 g; the spin mechanism achieves over a 1 mm displacement with a 3 mm eccentricity and an 8000 r/min rotation speed; the electrothermal mechanism achieves an 80.1 μm unlock distance under the active control of an external 11 V electrical signal.

Several tests are carried out to determine the designed device to meet the research objectives. The setback mechanism is verified to be able to unlock under the load of 15,000 g. The unlock test of the electrothermal mechanism demonstrates that the unlock displacement of the electrothermal mechanism reaches 71.25 μm, exceeding the minimum required displacement. The unlock test of the electrothermal mechanism and spin mechanism shows that the lock pin in the electrothermal mechanism completes the arming process coordinating with the spin mechanism at an input voltage of 11 V and a rotation speed of 8000 r/min. According to the function test, the MEMS S&A device is explosive proof in safe status while detonating in arming status.

The designed silicon-based MEMS S&A device realizes the integration of a setback mechanism, spin mechanism, electrothermal mechanism, and self-locking mechanism, overcoming the conflict between limited space and complex function, guaranteeing the explosive-proof ability as well as improving the level of miniaturization and integration of devices. It provides a research basis for the application of MEMS S&A devices in the next generation of weapons and related fields.

## Figures and Tables

**Table 1:** The designed size of setback mechanism.

Length l	Width w	Thickness h	Diameter d	Minimum Unlock Displacement w_max_
4000 μm	450 μm	100 μm	400 μm	200 μm

**Table 2:** Dimensions of S-shaped micro-springs.

Width b	Thickness h	Length l	Segments n	Radius R	Designed Stiffness *K*_1_	Theoretical Stiffness *K*_2_
40 μm	50 μm	600 μm	4	100 μm	21.7 N/m	19.9 N/m

**Table 3:** Time-delay mechanism-related parameters.

Width w	Thickness h	Length l	Number of Beams *n*	Degree *θ*	Length of Lever l_1_	Length of Lever l_2_
38 μm	50 μm	2150 μm	3	3°	100 μm	900 μm

**Table 4:** Calibration test data of the Machete Hammer tester.

Serial	The Number of Teeth	Voltage/mV	Overload/m·s^−2^
1	6	194	9593
2	7	315	15,577
3	8	420	20,769
4	9	525	25,962
5	10	680	33,628
6	11	840	41,540
7	12	990	49,955
8	14	1100	54,395

**Table 5:** Electrothermal actuator test data.

Voltage/V	Current/A	Displacement/μm	Output Displacement Curve
1	0	—	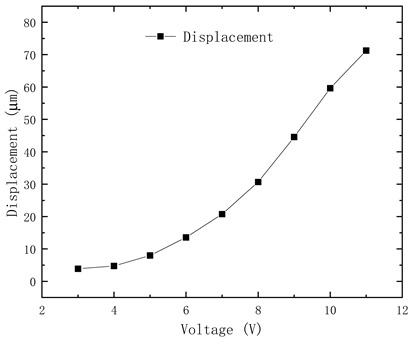
2	0.01	—	
3	0.01	3.87	
4	0.02	4.73	
5	0.03	7.97	
6	0.04	13.53	
7	0.05	20.74	
8	0.06	30.65	
9	0.06	44.57	
10	0.07	59.63	
11	0.07	71.25	

## Data Availability

The original contributions presented in the study are included in the article, further inquiries can be directed to the corresponding author.
